# Synthesis of Hierarchical MOR-Type Zeolites with Improved Catalytic Properties

**DOI:** 10.3390/molecules26154508

**Published:** 2021-07-27

**Authors:** Zeinab Mcheik, Ludovic Pinard, Joumana Toufaily, Tayssir Hamieh, T. Jean Daou

**Affiliations:** 1 Axe Matériaux à Porosité Contrôlée (MPC), Institut de Science des Matériaux de Mulhouse (IS2M), UMR 7361, CNRS, University of Haute Alsace (UHA), F-68093 Mulhouse, France; zeinab.mcheik@univ-poitiers.fr; 2 University of Strasbourg (Unistra), F-67000 Strasbourg, France; 3 Institut de Chimie des Milieux et Matériaux de Poitiers, UMR 7285 CNRS, 4 Rue Michel Brunet, Bâtiment B27, CEDEX 09, 86073 Poitiers, France; 4 Laboratory of Materials, Catalysis, Environment and Analytical Methods Faculty of Sciences, Section I, Lebanese University Campus Rafic Hariri, Hadath, Lebanon; joumana.toufaily@ul.edu.lb (J.T.); tayssir.hamieh@ul.edu.lb (T.H.)

**Keywords:** zeolites, hierarchical zeolites, mordenite, *n*-hexane cracking

## Abstract

Hierarchical MOR-type zeolites were synthesized in the presence of hexadecyltrimethylammonium bromide (CTAB) as a porogen agent. XRD proved that the concentration of CTAB in the synthesis medium plays an essential role in forming pure hierarchical MOR-type material. Above a CTAB concentration of 0.04 mol·L^−1^, amorphous materials are observed. These hierarchical mordenite possess a higher porous volume compared to its counterpart conventional micrometer crystals. Nitrogen sorption showed the presence of mesoporosity for all mordenite samples synthesized in the presence of CTAB. The creation of mesopores due to the presence of CTAB in the synthesis medium does not occur at the expense of zeolite micropores. In addition, mesoporous volume and BET surface seem to increase upon the increase of CTAB concentration in the synthesis medium. The Si/Al ratio of the zeolite framework can be increased from 5.5 to 9.1 by halving the aluminum content present in the synthesis gel. These synthesized hierarchical MOR-type zeolites possess an improved catalytic activity for *n*-hexane cracking compared to large zeolite crystals obtained in the absence of CTAB.

## 1. Introduction

Zeolites have a rigid framework structure with pores constrained in their sizes at the molecular level [1,2,3,4]. They exhibit attractive features that are superior to those of silica, such as their unique pores and their various framework compositions accessible by changing the Si/Al ratio or by substituting silicon atoms with atoms other than aluminum such as germanium or phosphorus. Zeolites also allow incorporating various metal cations in their micropores or external surface by cationic exchange or impregnation, allowing their use in different catalytic applications [5,6,7,8]. Unfortunately, when used in some catalytic reaction, conventional zeolites with micron-size particles have shown diffusion limitations (high diffusion path length) that can generate a deactivation of acid sites due to the retention of carbonaceous compounds [9,10,11,12]. Several studies have shown that additional larger pores (usually mesopores) can overcome possible diffusion/transport limitations of the smaller micropores of the zeolite [9,13,14].

Hierarchical zeolites can be obtained with different approaches: (i) Post synthesis treatment using either a destructive (top-down) approach, which consists of the dealumination or desilication of large zeolite crystals to create mesopores and macropores, or a constructive (bottom-up) approach by assembling nanocrystals and thus creating hierarchical porosity [9,15] and (ii) one-shot synthesis using hard template strategy (use of carbon nanotubes or polymers or silica beads as reactors) [16,17,18,19,20,21,22,23] or a soft templating strategy. In this case, organic additives are added to the medium generally used for the zeolite synthesis, like organosilanes [24], organic surfactant agents (CTAB, etc) [25], or bifunctional organic agents. These can direct the synthesis of zeolites and inhibit crystal growth, creating nanosheets or nanosponges zeolite materials [26,27,28,29,30,31].

Among all the zeolites, mordenite (MOR-type) zeolites possess remarkable properties that make them effective industrial catalysts in a variety of reaction (isomerization, carbonylation, hydrodeoxygenation, biomass conversion [32]), and selective adsorbents (electrochemical detection, adsorption of H_2_ and CO_2_, etc [32]). These properties give them excellent thermal stability since their amorphization occurs only above 800 °C [33]. The framework of MOR is relatively dense (17.2 T elements/1000 Å^3^) with two interconnected channel systems. The first is defined by a 12 member ring (MR) pore opening along the crystallographic c axis’s direction, with a pore size of 6.5 × 7.0 Å. The second channel system has a pore opening delimited by eight T elements (8 MR) of 2.6 × 5.7 Å. Side pockets, oriented in the crystallographic axis b, have a pore opening delimited by eight T elements (8 MR) of 3.4 × 4.8 Å. The first synthesized mordenite zeolite had Si/Al ratios close to the natural one (Si/Al = 5). Later, several silicon-rich mordenite MOR zeolites were synthesized [34,35]. The introduction of organic bases in the synthesis medium allowed obtaining mordenite with SiO_2_/Al_2_O_3_ ratios from 20 to 40 [36]. Recently, several studies were devoted to the synthesis of hierarchical MOR-type zeolite with reduced diffusion path length [37,38,39,40,41,42,43]. These strategies consisted on the use of sacrificial templates such as organosilanes [37,38,39] or hexadecyltrimethylammonium bromide (CTAB) [40,41].

In this paper, an innovative, facile, and direct approach is used to elaborate hierarchical MOR-type with reduced diffusion path length and improved catalytic properties. This approach consists of the addition to the synthesis mixture of CTAB as a meso-porogen agent without using an organic structure-directing agent to elaborate zeolite.

The obtained hierarchical-zeolites MOR are fully characterized. Then their catalytic properties will be evaluated using a model reaction consisting of *n*-hexane cracking.

## 2. Results and Discussions

Our main objective is to generate pure phase hierarchical MOR-type zeolites with reduced diffusion length and improve catalytic properties via the use of a single organic porogen (CTAB). The synthesis conditions (concentration of CTAB in the starting gel, temperature, and duration of the hydrothermal treatment) are optimized to obtain these hierarchical materials with different textural properties, morphologies, and crystal size.

The reproducible operating conditions used for the synthesis (molar composition of the gel, temperature, and duration of the hydrothermal synthesis) in addition to their textural properties are compared with those of sample synthesized in the absence of CTAB (0.033 S_0_^130/7^) and summarized in Table 1.

### 2.1. Effect of the Molar Concentration of CTAB on the Textural Properties

**Figure 1:**
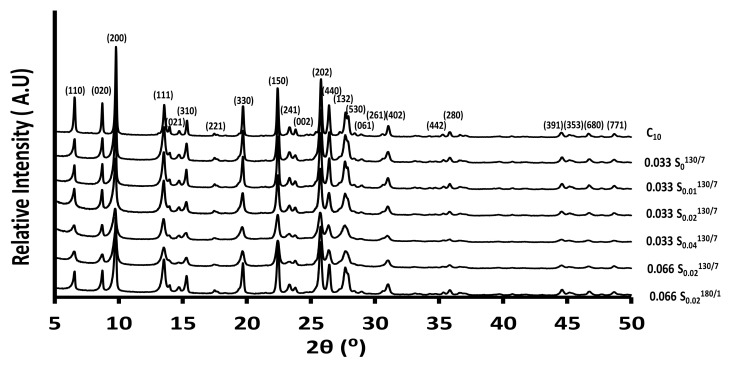
Wide-angle X-ray diffraction of the zeolites synthesized in the absence or presence (hierarchical zeolites) of CTAB.

The crystallinity and purity are checked by XRD. All samples are previously calcined before analyses. Regardless of the used synthesis protocol, only MOR crystalline phase is detected. Indeed, all the diffraction peaks observed are indexable and characteristic of the MOR zeolite (Figure 1). However, it should be noticed that with the increase of the molar concentration of CTAB in the reaction medium under the same conditions at 130 °C as shown in Figure 1 from the XRD patterns of samples 0.033 S_0_^130/7^, 0.033 S_0.01_^130/7^, 0.033 S_0.02_^130/7^, 0.033 S_0.04_^130/7^, enlargement and a decrease in peak intensities are observed compared to the sample 0.033 S_0_^130/7^ synthesized in the absence of CTAB. This is a characteristic of a reduction in zeolite crystallite size and a slight decrease in crystallinity. The phenomena is more pronounced for 0.033 S_0.04_^130/7^ sample synthesized with the highest amount of CTAB in the synthesis medium. No crystallized phase is detected above this amount of CTAB in the starting synthesis medium (only amorphous content).

The values related to diffusional path lengths in all the materials are the average interval of the height (L) (present in Table 1).

**Figure 2:**
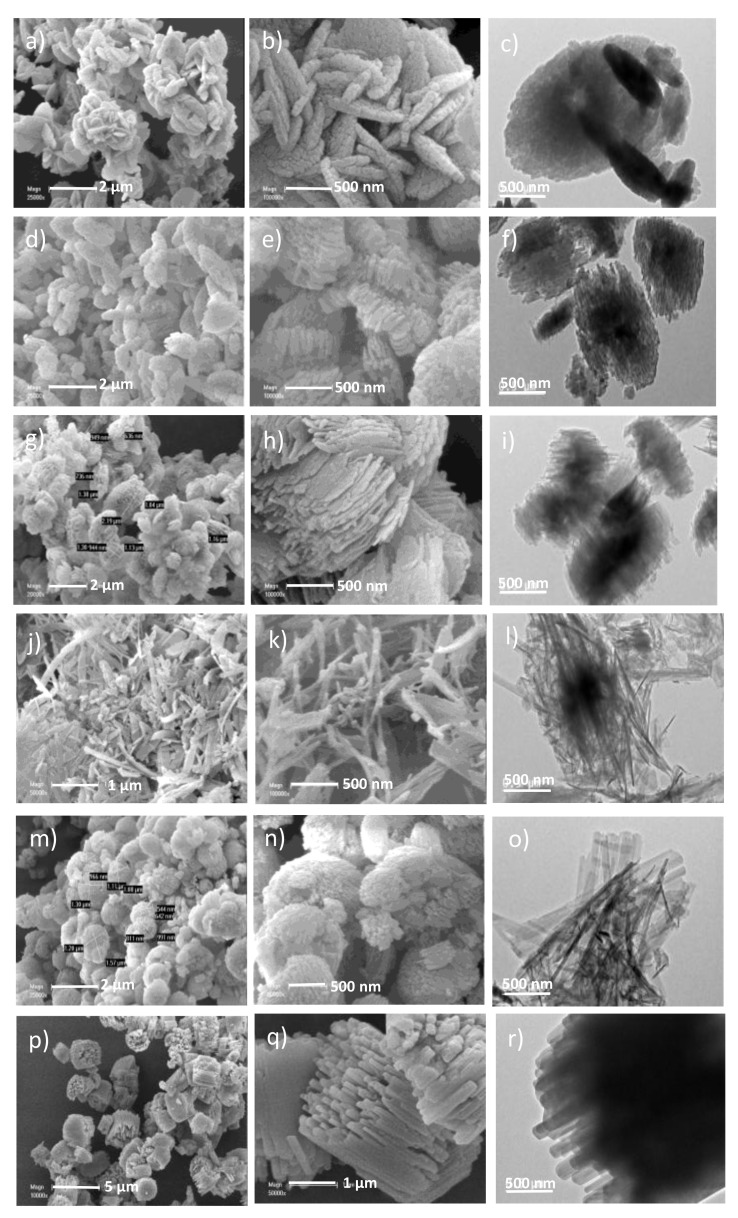
SEM and TEM images: 0.033 S_0_^130/7^ (**a**,**b**,**c**); 0.033 S_0.01_^130/7^ (**d**,**e**,**f**); 0.033 S_0.02_^130/7^ (**g**,**h**,**i**); 0.033 S_0.04_^130/7^ (**j**,**k**,**l**); 0.066 S_0.02_^130/7^ (**m**,**n**,**o**) and 0.066 S_0.02_^180/1^(**p**,**q**,**r**).

Moreover, optimization of the molar concentration of CTAB in starting gel provides an evolution in morphology and size of the crystals (0.033 S_0.01_^130/7^, 0.033 S_0.02_^130/7^_,_ 0.033 S_0.__04_^130/7^), which is displayed in SEM and TEM images (Figure 2). This optimization gives rise to inter-grown nanosheets or nanosticks composing hierarchical particles instead of platelet particles (0.033 S_0_^130/7^), obtained in the absence of CTAB (Figure 2). The dimensions of platelet particles range between 0.4 and 1.8 µm in length, 0.2–1.1 µm in width, and 0.04–0.09 µm in thickness. Indeed, the addition of CTAB to the synthesis mixture presents at the beginning at a low concentration (0.033 S_0.01_^130/7^) leads to a generation of hierarchical particles (see particle size in Table 1) composed of inter-grown nanosticks with a length varying from 115 to 225 nm in length and a width going from 21 to 45 nm. If the amount of CTAB is doubled (0.033 S_0.02_^130/7^), the synthesis gives rise to hierarchical particles (Table 1) composed of inter-grown nanosheets instead of nanosticks. The highest concentration of CTAB (0.033 S_0.04_^130/**7**^ sample) in the synthesis medium gives rise to distinct nanosticks with a length varying from 156 to 1800 nm in length and a width ranging from 9 to 115 nm. These observations corroborate the decrease of crystallite size observed from XRD patterns. CTAB can display a self-assembly effect for the synthesis hierarchical MOR-type zeolites. Indeed, the cationic head group of CTAB promotes the assembly with the negatively charged zeolite nucleus. This good affinity between the two species is responsible for the crystal growth inhibition and the formation of large pores.

The amount of CTAB in the final zeolitic material can be deduced from thermogravimetric analyses. Taking into account the amount of CTAB present in the starting synthesis medium, we are able to say that not all the CTAB is used for the creation of additional porosity in the zeolite.

**Figure 3:**
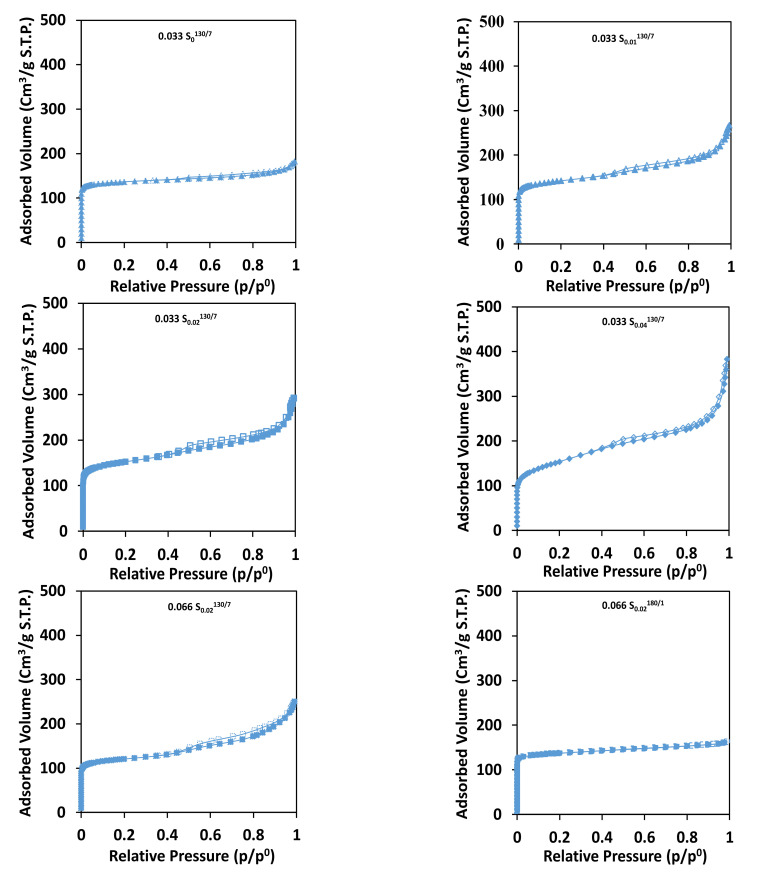
N_2_ adsorption (solid symbols) and desorption (open symbols) isotherms done at −196 °C of the calcined zeolites obtained in the absence or in the presence of CTAB.

The nitrogen adsorption-desorption isotherms at −196 °C of the calcined materials are shown in Figure 3. The nitrogen adsorption-desorption isotherms are of type I at low relative pressure as expected for a microporous material and IV isotherms at high relative pressures (for 0.033 S_0.01_^130/7^, 0.033 S_0.02_^130/7^_,_ and 0.033 S_0.04_^130/7^ samples). However, for the three samples synthesized CTAB, some interparticle mesoporosity is also clearly observed (type II at high *p*/*p*° (above 0.9)). The textural properties are reported in Table 1.

A microporous volume of 0.20 cm^3^ g^−1^ was calculated in the case of (0.033 S_0_^130/7^) respectively, which is the expected microporous volume for a well-crystallized MOR-type zeolite [44]. The addition of CTAB to the synthesis gel does not affect the microporous volume of samples 0.033 S_0.01_^130/7^, 0.033 S_0.02_^130/7^, and 0.033 S_0.04_^130/7^ which exhibit the same order of microporous volume (0.18–0.21 cm^3^/g) as the sample 0.033 S_0_^130/7^ synthesized in the absence of CTAB, indicating that the creation of mesopores does not occur at the expense of micropores. In addition, mesoporous volume and BET surface seem to increase upon the increase of CTAB concentration in the synthesis medium (Figure 3).

The acidic properties of zeolites were characterized by XRF, ^27^Al MAS NMR, and pyridine adsorption followed by FTIR. Their main parameters are summarized in Table 2. The addition of CTAB to the synthesis medium seems to increase the overall Si/Al ratio of the obtained samples slightly (from 8 without CTAB to 9.1 for sample 0.033 S_0.04_^130/7^), as shown in Table 2 for XRF analyses.

**Figure 4:**
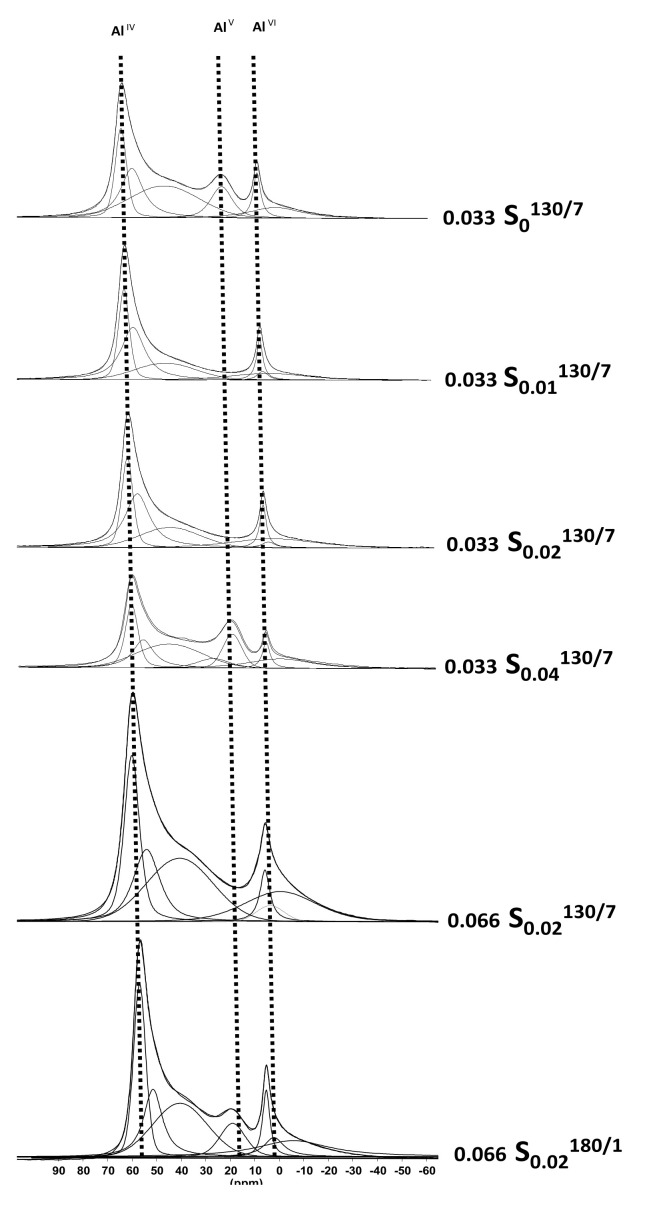
^27^Al-MAS-NMR spectra of the calcined zeolites.

^27^Al MAS-NMR is known to be applied to determine and quantify the different coordination states of this nucleus.^27^Al MAS-NMR spectra (Figure 4) of the synthesized MOR samples (0.033 S_0_^130/7^, 0.033 S_0.01_^130/7^, 0.033 S_0.02_^130/7^ and 0.033 S_0.04_^130/7^) show two or three signals. One is characteristic of the resonance of tetracoordinated aluminum atoms, another corresponds to the resonance of pentacoordinated aluminum atoms, and the other is characteristic of the resonance of hexacoordinated aluminum. The tetracoordinated aluminum atom Al^IV^ resonates at a chemical shift of 54 ppm. The pentacoordinated aluminum atoms Al^V^ resonate at a chemical shift of 12 ppm, and Al^VI^ hexacoordinated aluminum atoms at a chemical shift around 0 ppm. The percentage of each aluminum species (Al^IV^ as intra-framework and Al^V^ and Al^VI^ as extra-framework aluminum “EFAL”) is quantified by integrating the signals corresponding to each of these species. The quantitative analysis of each of these families by ^27^Al MAS-NMR combined with XRF results allows the calculation of the Si/Al ratio of the framework and to determine the number of extra framework aluminum “EFAL” per zeolite unit cell. The number of extra-framework Al atoms per unit cell seems to vary from 1 to 2, but seems not directly related to the CTAB concentration in the synthesis medium.

**Figure 5:**
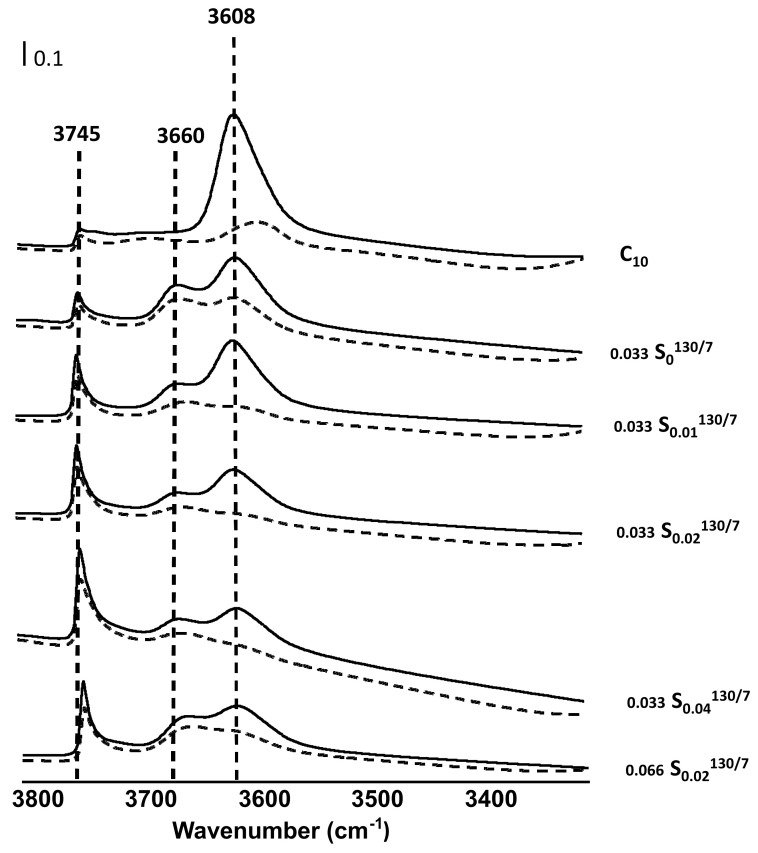
IR Spectra of the synthesized MOR-type zeolites before (full lines) and after (dashed lines) pyridine adsorption at 150 °C.

Figure 5 compares the hydroxyl stretching vibration region of IR spectra before (dotted line) and after (full line) adsorption of pyridine at 150 °C of the catalyst series. MOR synthesized without meso-porogen agent (0.033 S_0_^130/7^) exhibits after calcination an asymmetric band at 3608 cm^−1^ assigned to the bridging hydroxyl groups (i.e., acidic hydroxyl groups) and bands at 3745 and 3660 cm^−1^ due to stretching vibrations of external silanols Si-OH, and OH linked to extra-framework aluminum species (EFAL), respectively. The adsorption of pyridine leads to a partial decrease of the intensity of the 3608 and 3655 cm^−1^ bands. The incomplete neutralization of bridged OH groups by pyridine indicates the presence of inaccessible OH groups and that only a small proportion of hydroxylated EFAL have acid properties. EFAL species partially block the access of the pyridine to BAS; only 65% of the acidic hydroxyl groups are neutralized by the organic probe (Table 2). It is worth mentioning that the pore blocking takes place only with the pyridine (K_D_ = 5.6 Å) and not with nitrogen (K_D_ = 3.6 Å), suggesting that the size of the EFAL species within the main channel are smaller than (7−3.6 = 3.4 Å). The theoretical proton as BAS drawn from the accessibility degree and ^27^Al MAS-NMR is almost two times higher than that measured with pyridine. 

This discrepancy means that the hydrothermal conditions used for the synthesis result in 50% of the framework aluminum sites being distorted. The concentration of the Lewis acid site is rather limited compared to the number of EFAL species, which means that only a small proportion of them have Lewis acid properties.

The addition of CTAB in the synthesis gel leads to a slight increase of the silanol bands. When the molar concentration of CTAB increases in the synthesis medium from 0 to 0.04 (0.033 S_0.01_^130/7^, 0.033 S_0.02_^130/7^, and 0.033 S_0.04_^130/7^ catalysts), an improvement of the accessibility is observed up to 90%. Nevertheless, the percentage of distorted aluminum species present in the zeolite framework is always important regardless of the CTAB amount added to the gel, ranging from 40 to 55%. The high accessibility of the BAS despite an increased number of EFAL species (1.0–2.0) suggests that most of them are located on the generated mesopores. The lower the concentration of Brønsted acid sites (For the sample obtained in the absence of CTAB, the value was corrected with the degree of accessibility). CTAB allows the generation of intracrystalline mesopores to the extent of a part of the Brønsted acidity.

### 2.2. Effect of Aluminum Content in the Synthesis Medium

The amount of aluminum reagent introduced in the synthesis was doubled to increase the number of aluminum atoms in the zeolite framework (the molar concentration of Al_2_O_3_ is increased from 0.033 to 0.066 mol·L^−1^). A pure MOR-type phase is obtained for 0.066 S_0.02_^130/7^ samples, as shown in Figure 1. Still, the intensities of the XRD peaks are lower than the XRD peaks of 0.033 S_0.02_^130/^^7^ synthesized in the same conditions but with two less aluminum content in the synthesis medium, indicating a lower crystallinity for the 0.066 S_0.02_^130/7^ sample. The addition of a higher amount of aluminum in the synthesis medium seems to decrease the crystallization kinetics. This phenomenon was already observed in previous works [45]. The hydrothermal temperature is increased from 130 °C to 180 °C for one day instead of 7 days to increase sample crystallinity. Pure MOR-type phase was also obtained for 0.066 S_0.02_^180/1^ with improved crystallinity compared to sample 0.066 S_0.02_^130/7^, as shown from XRD patterns in Figure 1. Indeed, XRD peaks with higher intensities compared to those of 0.066 S_0.02_^130/7^ are observed.

SEM and TEM images of sample 0.066 S_0.02_^130/7^ display agglomerates varying from 0.3 to 1.9 µm in length, from 0.3 to 1.4 µm in width, and from 0.4 to 0.7 µm in length thickness. These agglomerates are composed of agglomerated MOR-type sticks, which possess a length ranging between 300 to 1900 nm and a width ranging between 10 to 116 nm. An increase in the crystal (sticks) size is observed once the aluminum content is doubled in the synthesis medium (Table 1, the comparison between crystal size of samples 0.066 S_0.02_^130/7^ and 0.033 S_0.02_^130/7^). Increasing the hydrothermal treatment temperature increases the agglomerates and crystal size (0.066 S_0.02_^180/1^) compared to the 0.066 S_0.02_^130/^^7^ sample, but similar morphologies are still observed (Figure 2). The nitrogen adsorption-desorption isotherms at −196 °C of the calcined materials are shown in Figure 3. The nitrogen adsorption-desorption isotherms of 0.066 S_0.02_^130/7^ and 0.066 S_0.02_^180/1^ samples shown in Figure 3 are of type I at low relative pressure as expected for a microporous material and IV isotherms at high relative pressures. Moreover, an increase in the microporous volume is observed by increasing the hydrothermal treatment temperature, which corroborates with the increase of crystallinity observed from XRF patterns. On the contrary, a drastic decrease in the mesoporous volume is observed in the case of 0.066 S_0.02_^180/1^ compared to 0.066 S_0.02_^130/7^ (Figure 3).

The total Si/Al ratio deduced from XRF of both MOR samples synthesized with a higher amount of aluminum in the starting gel synthesis is around 5.5 instead of 8.5 when aluminum is 2-fold less (0.033 S_0.02_^130/7^) (Table 2). 

Figure 4 displays ^27^Al-MAS NMR spectra of both synthesized samples (0.066 S_0.02_^130/7^ and 0.066 S_0.02_^180/1^). One major resonance is detected around 54 ppm corresponding to tetrahedrally coordinated aluminum Al(OSi)_4_, as expected for a MOR-type zeolite. Additional signals corresponding to extra-framework aluminum can also be observed: around 0 ppm attributed to Al^VI^ hexacoordinated aluminum atoms for both samples and approximately 17 ppm attributed to Al^V^ pentacoordinated aluminum atoms (only for 0.066 S_0.02_^180/1^). The proportion of EFAL increases while increasing the aluminum content in the synthesis medium (from 1.3 to 2.3).

Doubling the concentration of aluminum in the synthesis gel allows more Al to be inserted into the MOR framework and also a large amount of EFAL species (>2.3) (Table 2). Despite a more significant amount of the extra and framework aluminum species, the concentration of the Brønsted and Lewis acid sites is lower or, in the best case, similar. This result is not due to pore blocking by the EFAL species but suggests that the additional aluminum in the zeolite framework is distorted. EFAL species are located outside the micropores and do not have Lewis acidity properties.

### 2.3. n-Hexane Cracking

**Figure 6:**
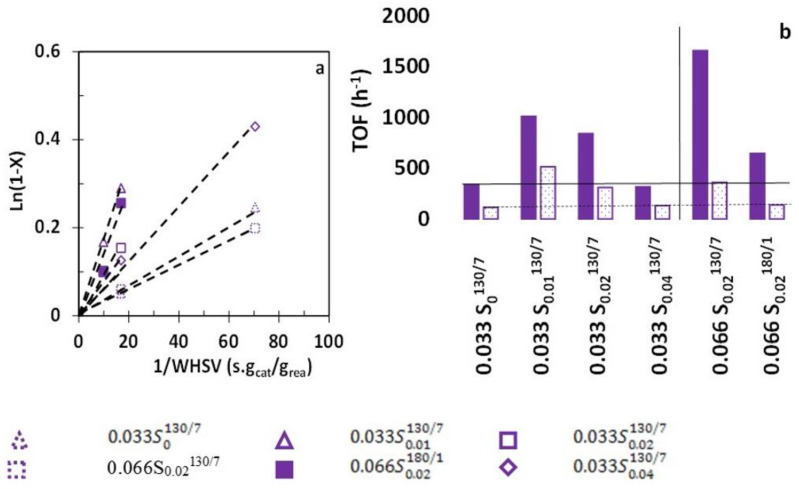
*n*-Hexane cracking: (**a**) Test for the first-order rate equation, (**b**) Turnover frequencies per theoretical (empty bar) and probed (full bar) Brønsted acid site.

*n*-Hexane cracking is carried out at 540 °C at atmospheric pressure under nitrogen flow with a *N*_2_/*n*-hexane molar ratio of 9. Figure 6 displays Ln (1−X) as a function of 1/WHSV obtained after 1 min. Ln (1−X) vs. contact time gives a straight line through the origin, indicating a pseudo-apparent-first-order. This order is consistent with the n-monomolecular cracking mechanism.

The bar chart in Figure 6b compares the turnover frequencies per Brønsted acid site probed by pyridine. TOF increases when CTAB is added to the synthesis medium and is much higher when the aluminum concentration is doubled. However, a higher concentration (0.04 mol·L^−1^) or high temperature is detrimental to the activity per site. But the kinetic diameter of *n*-hexane (4.3 Å) is lower than that of pyridine (5.6 Å); hence some acid sites inaccessible to the pyridine can be accessible to the linear paraffin. Thus, turnover frequency should be calculated from the theoretical acidity drawn from ^27^Al MAS-NMR. Regardless of the TOF calculation method used, the addition of CTAB to the synthesis medium improves the catalytic activity.

## 3. Materials and Methods

### 3.1. Mordenite Synthesis 

The protocol of synthesis of hierarchical MOR-type zeolite was adapted from the protocol published by Yuan et al. [41]. This protocol requires the use of several organic additives (tetraethyl ammonium hydroxide (TEAOH), CTAB, sodium dodecylbenzene sulfonate surfactant (SDBS), etc) whereas, in our protocol, only CTAB is used as a porogen agent. The gel composition and the synthesis conditions (hydrothermal treatment and time) were also optimized in order to obtain pure and well crystallized hierarchical MOR-type zeolites. The gel molar composition was as follows: SiO_2_: w Al_2_O_3_: 0.25 Na_2_O: 40 H_2_O: x CTAB.

Several types of chemicals were employed in this synthesis, including NaOH (>97%, Carlo Erba, Val-de-Reuil, France), NaAlO_2_ (43.95% Na_2_O, 56% Al_2_O_3_, 0.05% Fe_2_O_3_, Sigma-Aldrich, Saint Louis, MO, USA), colloidal silica (30 wt.% SiO_2_, Sigma-Aldrich), and hexadecyltrimethylammonium bromide (CTAB, 98%). The preparation is conducted by dissolving a solution of CTAB mixed with aqueous sodium hydroxide solution, followed by the addition of NaAlO_2_ then colloidal silica while maintaining a high, stirring speed. To keep over the homogeneity of the gel, magnetic stirring was maintained for three hours followed by hydrothermal treatment in a Teflon^®^ lined stainless steel autoclave at x °C for y days under 50 rpm rotation in an oven equipped with mechanical rotation.

The precipitated product was then obtained by centrifugation and washed several times with deionized water, dried at 80 °C overnight, then calcined under air at 550 °C for 14 h with a temperature ramp of 1 °C/ min, in order to remove the organic porogen. The calcined products were then protonated with an NH_4_NO_3_ aqueous solution (1 mol·L^−1^) (1 g of the calcined sample in 35 mL solution). This exchange was done for one hour under stirring at 80 °C and was repeated three times to increase the cationic exchange ratio in favor of proton.

The following nomenclature is used for the obtained samples w S_x_ ^y °C/ z Days^. W stands for the number of Al_2_O_3_ moles present in the starting synthesis mixture, S for the use of Organic porogen, and X is the number of moles of organic porogen (CTAB) present in the starting gel, Y is the crystallization temperature, and Z the crystallization duration. For example, 0.033 S_0.02_^130/7^ corresponds to the MOR hierarchical sample synthesized at 130 °C for seven days in the presence of 0.033 moles of Al_2_O_3_ and 0.02 moles of CTAB in the starting synthesis mixture.

### 3.2. Characterization

The crystalline structure of synthesized Mordenite zeolite was determined by powder X-ray diffraction (XRD) on a MPD X’Pert Pro diffractometer (PANalytical, Limeil-Brévannes, France) operating with Cu Kα radiation (λ = 0.15418 nm) equipped with an X’Celerator real-time multiple strip detector (active length = 2.122° 2θ). The measurements were carried out at 22 °C on an angular range 2θ varying from 3 to 50°, with a 2Ө angle step of 0.017° and a time step of 220 s.

The morphology, homogeneity, and particle sizes of the obtained samples were performed with a scanning electron microscope (SEM; XL30 FEG, Philips, FEI-Thermo Fisher Scientific, Eindhoven, Netherlands)) working at 7 kV accelerating voltage and by transmission electron microscopy (TEM; model CM200, Philips, Eindhoven, Netherlands), under an acceleration voltage of 200 kV, with a point-to-point resolution of 0.3 nm.

The N_2_ adsorption/desorption isotherms were measured at −196 °C with a 2420 ASAP (Micromeritics, Merignac, France). Prior to this measurement, the calcined zeolite was degassed under vacuum for 15 h at 300 °C. The adsorption branches were used to calculate the surface area and the size of the mesopores distributions. The surface area was calculated from the Brunauer-Emmett-Teller (BET) equation using the following range 0.05 < *p*/*p*^0^ < 0.3. The micropores volume (Vmicro) was determined using the method suggested by Galarneau [42,43].

The molar ratio of Si/Al was determined with an X-ray fluorescence spectrometer (Magic-X, Philips) equipped with a 3 kW power tube (with a rhodium anode). 200 mg of each of the zeolite samples studied were crushed with a mortar and then transformed into a cylindrical pellet after applying a pressure of 4 tons (using a hydraulic press) for 2 min to analyze them.

Rotating nuclear magnetic resonance (NMR) at magic angle ^27^Al (I = 5/2) was performed using an Avance II 400 spectrometer (Bruker, Wissembourg, France) operating at B_0_ = 9.4 T (Larmor frequency ν_0_ = 104.2 MHz) equipped with a dual-channel 4 mm Bruker probe. The materials were spun at 12 kHz, and the free inductance disintegrations (FIDs) were collected with a rf pulse π/12 (0.5 μs), and a recycle time of 1 s. The measurements were performed with [Al(H_2_O)_6_]^3+^ as a standard external reference.

Infrared spectroscopy (FTIR) of pyridine adsorption was done to study the strength, concentration, and the nature of acidity of synthesized samples by employing a bench using a Nicolet 6700 spectrometer (Thermo Scientific, Waltham, Massachusetts, USA) that can scan a range of wavenumbers from 1100 to 4000 cm^−1^ with a resolution of 2 cm^−1^. The sample (20 mg) is compressed using a hydraulic press (0.5 tons.cm^−2^) to form a wafer. Then in a Pyrex cell equipped with a vacuum system, the wafer is placed, and the system is then degassed for 1 h at 500 °C under nitrogen or hydrogen flow (100 mL.min^−1^) then cooled to 200 °C. A primary and then secondary vacuum is performed for 1 h, and finally, the pyridine is sent over the catalyst (1.5 mbar) for 5 min at 150 °C and evacuated for a period of 1 h. A first IR spectrum is recorded before pyridine adsorption and others after desorption of pyridine at increasing temperatures ranging from 150 to 450 °C. The quantities of pyridine adsorbed at the Brønsted [PyH^+^] and Lewis [PyL] sites are estimated from the integration of the band at 1545 and 1454 cm^−1^ by applying predetermined extinction factors. 

### 3.3. Catalyst Tests

*n*-Hexane cracking was carried out in a tubular glass fixed-bed microreactor under plug flow conditions. Before the reaction, samples (0.2–0.4 mm particle size) were pretreated overnight at 540 °C under a flow of dry nitrogen. The operating conditions were applied as listed below: 540 °C, 0.1 MPa, N_2_/n − C_6_ = 9, and various contact times. The reaction products were sampled during one h in a ten port-valve, then analyzed using a gas chromatograph (GC, Agilent, Les Ulis, France) equipped with a flame ionization detector (FID). A 50 m Cp-Al_2_O_3_/Na_2_SO_4_ capillary column of 0.32 mm internal diameter and a 60 m BR wax column of 0.25 internal diameter were used [46].

## 4. Conclusions

The introduction of mesoporosity in MOR-type zeolite was a success thanks to the introduction in the synthesis medium of CTAB as a porogen agent. Two critical parameters have been emphasized: the concentration of CTAB and aluminum content in the synthesis media. Indeed, XRD proved that the concentration of CTAB in the synthesis medium plays an essential role in forming pure hierarchical MOR-type material. Above a CTAB concentration of 0.04 mol·L^−1^, amorphous materials are observed. Nitrogen sorption showed the presence of mesoporosity for all mordenite samples synthesized in the presence of CTAB. This additional porosity did not occur at the expense of zeolite micropores. Mesoporous volume and BET surface area seem to increase upon increasing CTAB concentration in the synthesis medium. The increase of aluminum content in the synthesis medium allowed the preparation of hierarchical zeolite with higher aluminum content in their frameworks. These synthesized hierarchical materials showed an improved catalytic activity towards *n*-hexane cracking. This simple approach paves a new way for obtaining hierarchical zeolite materials of controlled porosity by a direct approach for specific catalytic applications.

## Figures and Tables

**Table 1:** Textural properties of the obtained zeolites.

Catalyst	[CTAB]	Particle Size ^a^	Shape & Crystal Size ^a^	V_micro_^b^	V_meso_^c^	S_BET_^d^
	mol·L^−1^	L × W × T µm × µm × µm	L × W nm × nm	cm^3^/g	cm^3^/g	m^2^/g
0.033 S_0_^130/7^	0	0.4–1.8 * 0.2–1.1 * 0.04 * 0.09	Platelet particles 0.4–1.8 × 0.2–1.1	0.20	0.06	446
0.033 S_0.01_^130/7^	0.01	0.4–1.8 * 0.2–0.9 * 0.15–0.34	Nanosticks 115–225 × 14–32	0.18	0.13	545
0.033 S_0.02_^130/7^	0.02	0.5–2.4 * 0.3–1.1 * 0.3–0.7	Nanosheets 300–700 × 14–32	0.20	0.15	574
0.033 S_0.04_^130/7^	0.04	Single sticks (see crystal size)	Nanosticks 156–1800 × 9–115	0.18	0.22	565
0.066 S_0.02_^130/7^	0.02	0.3–1.9 * 0.3–1.4 * 0.4–0.7	Nanosticks 300–1900 × 10–116	0.17	0.15	377
0.066 S_0.02_^180/1^	0.02	1.6–4.3 * 0.8–3.1 * 1.1–2.5	Sticks 1600–4300 × 56–198	0.20	0.04	506

^a^ Size and morphology of particles and crystals determined by SEM and TEM. ^b^ Determined with the corrected t-plot method [42,43]. ^c^ Calculated by subtracting microporous volume from the total volume; Mesoporous volume: V_meso_ = V_tot_ − V_micro_. * value calculated with Vtot determined at *p*/*p*° = 0.90 without interparticle porosity. ^d^ Specific surface area determined by using the Brunauer-Emmet-Teller (BET) method.

**Table 2:** Acidic properties of the synthesized MOR-type zeolites obtained in the absence or in the presence of CTAB.

Catalyst	Al^IV a^	Al^V a^	Al^VI a^	Si/Al ^b^	EFAL Per Unit Cell ^a^	[Na] ^b^ wt.%	Theoretical Acidity	[H^+^] ^c^	[L] ^d^	Acc ^c^
	%			Total			µmol·g^−1^			(%)
0.033 S_0_^130/7^	70	10	20	8.0	1.7	0	1270	434	88	65
0.033 S_0.01_^130/7^	80	0		8.5	1.0		1386	705	123	92
0.033 S_0.02_^130/7^	75		25		1.3		1259	466	120	
0.033 S_0.04_^130/7^	60	20	20	9.1	2.0	0.06	959	401	128	90
0.066 S_0.02_^130/7^	70	0	30	5.5	2.3	0	1748	381	146	95
0.066 S_0.02_^180/1^	65	10	25		2.7		1600	347	14	n.d

^a^ Site% determined by ^27^Al MAS-NMR. ^b^ Global Si/Al and wt.% of Na residual measured by XRF. ^c,d^ Brϕnsted and Lewis acidity measured by pyridine adsorbed at 150 °C. ^e^ Measured by the intensity of the hydroxyl group before and after the adsorption of pyridine at 150 ° C.

## Data Availability

The funders had no role in the design of the study; in the collection, analyses, or interpretation of data; in the writing of the manuscript, or in the decision to publish the results.

## Sample Availability

The samples are available from T.J.D.
